# Impact of Physical Activity on Ovarian Response: A Prospective Study Among In Vitro Fertilization Patients

**DOI:** 10.7759/cureus.98640

**Published:** 2025-12-07

**Authors:** Fatima Olaso, Rosario Mendoza, Iñaki Echeverria, Iker Malaina, Jone Ibañez, Jon Irazusta, Roberto Matorras

**Affiliations:** 1 Obstetrics and Gynecology Department, Human Reproduction Unit, Cruces University Hospital, Barakaldo, ESP; 2 Physical Education and Sports Department, University of the Basque Country, Leioa, ESP; 3 Mathematics Department, University of the Basque Country, Leioa, ESP; 4 Physiology Department, University of the Basque Country, Leioa, ESP; 5 Reproductive Medicine, IVI Bilbao, IVIRMA, Leioa, ESP

**Keywords:** assisted reproduction technology, infertility and art, introcytoplasmic sperm injection (icsi), in vitro fertilization (ivf), mature oocyte, oocyte quality, ovarian response, physical activity, physical exercise

## Abstract

Introduction

Scientific evidence has shown that regular physical activity (PA) is beneficial to health. However, no consensus has been reached on the association between PA and the success rates of assisted reproduction treatments. The purpose of the present study was to determine whether various levels of physical activity have an influence on ovarian response to controlled stimulation in ‘in vitro fertilization’ (IVF) or ‘intracytoplasmic sperm injection’ (ICSI) cycles, defined as number of retrieved and mature oocytes.

Methods

This prospective observational study included 617 infertile women undergoing IVF/ICSI cycles between January 2019 and October 2020. PA was assessed prior to the IVF cycle, using the International Physical Activity Questionnaire short form (IPAQ) and triaxial accelerometers. Patients were classified into three groups: low, moderate and high PA.

Results

Globally, the number of retrieved oocytes was similar in all three groups according to IPAQ (9.23 ± 7.72; 8.35 ± 5.57; 8.82 ± 6.38). Something similar happened with the number of mature oocytes (6.97 ± 5.99; 6.84 ± 4.85; 7.05 ± 5.61). PA did not influence the number of oocytes (retrieved or mature) in most clinical subgroups established (age, smoking, body mass index (BMI), anti-mullerian hormone (AMH)). However, in the subgroup 'endometriosis' the number of mature oocytes was significantly superior in high and moderate vs low PA (p=0.024). In the subgroup 'ovulatory disorders' there were also more mature oocytes in high and moderate vs low PA (p=0.038).

When performing the analysis according to accelerometer there were globally no significant differences between PA groups, nor in most clinical subgroups considered. Only in women with normal BMI, high PA was significantly associated with a greater number of collected (p=0.005) and mature oocytes (p=0.004).

Conclusion

Globally, PA had no influence on ovarian response in IVF cycles, defined as number of retrieved and mature oocytes. However, in certain clinical subgroups (endometriosis, ovulatory disorders, normal-BMI) high PA was associated with a superior ovarian response.

## Introduction

Lifestyle is considered to be the main determinant of an individual's health. Scientific evidence has shown that regular physical activity (PA) is beneficial to health. According to the World Health Organization, moderate PA is an effective way to lower the risk of many pathologic conditions, although its effect on female fertility is not well known [1].

There is solid evidence that both sedentary behaviour and excessive PA can be detrimental for female fertility. On one hand, sedentary lifestyle increases the risk of obesity, which is associated with menstrual and ovulatory disorders, higher miscarriage rate and lower pregnancy rate. On the other hand, high-performance sport involves an increased risk of neuroendocrine and reproductive dysfunction, which may interfere with the achievement of a pregnancy. This disruptive effect of elite-level sport on the menstrual cycle of female athletes has been widely described in scientific literature.

However, no consensus has been reached on the association between PA and the success rates of an assisted reproduction treatment. Indeed, assessment of PA is complex. Most published studies used self-administered questionnaires [2-8] and there are few clinical studies with objective evaluation of PA [9-11]. Meta-analyses published to date report a significant increase in clinical pregnancy rates in patients who performed regular PA prior to the IVF cycle [12-14], although it is unknown whether this is due to a better oocyte factor or to a superior endometrial receptivity. Moreover, the type, frequency, volume and intensity of an optimal intervention also remain unclear [14]. Clinical studies evaluating the effect of PA on ovarian response are scarce, although murine models suggest that PA may have a positive impact on it [15-17].

Therefore, the aim of the present study was to determine whether PA influences ovarian response in ‘in vitro fertilization’ (IVF) or ‘intracytoplasmic sperm injection’ (ICSI) cycles and to analyze the results in certain clinical subgroups: advanced maternal age, smoking, body mass index (BMI), low ovarian reserve and ovarian factor (endometriosis, ovulatory disorders). This article was previously presented as a poster at the European Society of Human Reproduction and Embryology (ESHRE) 39th Annual Meeting on June 26, 2023 [18].

## Materials and methods

The study population consisted of 617 infertile women undergoing IVF/ICSI cycles at the Human Reproduction Unit, Cruces University Hospital (Spain), between January 2019 and October 2020. PA was assessed one month before controlled ovarian stimulation using two methods: International Standardised PA Questionnaire (IPAQ), which assesses frequency, intensity and duration of PA in the last seven days (n=617 patients) [19] and triaxial accelerometer ActiGraph wGT3X-BT® (Ametris, Pensacola, FL, USA) (n=105 patients, included in the population of 617 who answered the IPAQ survey) [20].

All consecutive patients who were going to start an IVF cycle and met the inclusion criteria were asked to complete the survey; most of them accepted, forming the IPAQ group. Of these, 105 consecutive patients underwent PA assessment by means of an accelerometer between January and October 2020, forming the accelerometer group. Sample size was calculated by means of Granmo Datarus® software (Regicor, IMIM, Barcelona, Spain) [21].

Concerning the design, it was a single-centre prospective observational clinical study. Ethical approval was obtained from the Clinical Research Ethics Committee (CEIC E19/06) and informed consent was signed by all subjects before their participation.

Inclusion criteria were as follows: patients starting fresh IVF/ICSI cycle with own gametes, women aged 18 to 40 years, duration of infertility more than one year, anti-mullerian hormone (AMH) > 0.4 ng/mL, previous completion of no more than two cycles of ovarian stimulation for IVF/ICSI, BMI <35 kg/m², signed informed consent. Exclusion criteria were having started ovarian stimulation or not meeting any of the inclusion criteria. No preimplantation genetic testing for aneuploidy (PGT-A) cycles were included since, at the time of the study, it was not available at our center.

The following characteristics were collected for each patient: age, weight and height, etiology of infertility, smoking habit and AMH value. BMI was calculated as weight (kg)/height² (m²). The population's subgroups were defined as follows: advanced maternal age (women aged 37-40 years), smoking (any amount of tobacco), BMI (obese 30-34.9 kg/m², overweight 25-29.9 kg/m², normo-weight 18.5-24.9 kg/m²), low ovarian reserve (AMH <1 ng/mL), ovarian factor (endometriosis or ovulatory disorders).

Assessment of PA: International Physical Activity Questionnaire, short form (IPAQ)

The IPAQ is an international standardized and validated method to assess PA performed during the last seven days by means of a questionnaire. It has shown a good correlation with PA performed in the last weeks and in a typical week [19].

The IPAQ encompasses not only sport or recreational PA but also non-recreational PA (transport, work, housework, etc.). Since people generally follow a routine in their daily lives, reporting PA in the last seven days is usually fairly representative of that person's PA habits.

One month before ovarian stimulation, the patient was invited to fill in the short version of the IPAQ questionnaire, which collects frequency (in days per week), duration (in minutes per week) and intensity of PA performed in the last week. With regard to intensity of PA, the survey is structured in four groups: vigorous, moderate, walking and sedentary behaviour. Intensity of PA was estimated using metabolic equivalents of task (MET). 1 MET (3.5 ml O₂/kg/min) represents the energy expenditure at rest. For each PA level, the average MET was calculated using the compendium by Ainsworth, attributing 8 MET for vigorous PA, 4 MET for moderate PA and 3.3 MET for walking [22]. Using these measurements, the results of the short version of the IPAQ were calculated, obtaining a total PA score in MET-minutes/week, assessed by the sum of walking, moderate and vigorous MET-minutes.

By means of the IPAQ embedded software, women were classified according to their total PA scores into three levels: low, moderate and high PA. Participants were included at the moderate PA level if they performed a minimum of three days of vigorous PA of at least 20 minutes per day or a minimum of five days of moderate PA and/or walking of at least 30 minutes per day or a minimum of five days of any combination (walking, moderate or intense PA) reaching 600 MET-minutes/week. Women were included at the high PA level if they met one of the following criteria: to perform a minimum of three days of intense PA reaching at least 1500 MET-minutes/week or to perform seven days of any combination (walking, moderate or intense PA) of at least 3000 MET-minutes/week. Participants were included at the low PA level if they performed no PA or some PA but without meeting criteria for moderate or high categories.

Assessment of PA: triaxial accelerometer

A subgroup of consecutive patients (n=105), who previously completed the IPAQ survey, were provided with an ActiGraph wGT3X-BT® triaxial accelerometer (Ametris, Pensacola, FL, USA) to objectively assess PA for seven consecutive days before starting ovarian stimulation (device initialization preset: frequency 100 Hz, Epoch 60s, normal filter option) between January and October 2020 [20]. Patients wore the accelerometer on the right hip and data were collected during 16 hours a day. Wearing the device for at least four days was considered as a valid week, and at least 10 hours of activity was considered as a valid day. Non-wear time was established at 90 minutes. It included periods when the subject was asked not to wear the device (sleeping, bathing, swimming) or forgot to wear the device, and periods of inactivity, when the device was worn but no PA was performed for more than 90 minutes [23-25]. The acceleration data were recorded in the activity unit ‘counts’.

ActiLife 6® software (Ametris, Pensacola, FL, USA) was used to process data, using algorithms to convert acceleration data into PA data based on anthropometric characteristics [20]. A positive correlation has been established between the number of counts, oxygen consumption and MET, making it possible to calculate PA intensity using validated cut-off points. Those described by Sasaki in 2011 were used: light PA < 2689 counts (1.5-3 MET); moderate PA: 2690-6166 counts (3-6 MET); vigorous PA: 6167-9642 counts (6-9 MET); very vigorous PA ≥9643 counts (>9 MET) [26].

Accelerometers collected more than 50 PA parameters. Many of them were difficult to interpret clinically, so four representative PA variables were selected for further comparative analysis: total MET (MET-minutes/week), total ‘counts’ (counts-min/week), number of steps per day and sedentary behaviour (hours/day).

Treatment protocol

Patients underwent controlled ovarian stimulation with a GnRH antagonist protocol, and recombinant and/or urinary gonadotrophin doses between 225-300 IU/day were used. Follicular growth was monitored by vaginal ultrasound and by determination of serum oestradiol and progesterone levels. When three follicles reached 18.5 mm in diameter, final follicular maturation was triggered with hCG 250mcg or GnRH agonist (triptorelin bolus 0.2 ml) if there was a risk of hyperstimulation. 36 hours later, ultrasound-guided oocyte pick-up was scheduled. Assessment of mature oocytes (metaphase II) was based on morphological parameters.

The primary outcome was the number of mature oocytes (identified by the presence of the first polar body). The secondary outcome was the number of retrieved oocytes.

Statistical analysis

Data were analyzed using SPSS Statistics 18 (SPSS Inc., Chicago, Ill., USA). The Kolmogorov-Smirnov test was performed to verify normality. Since most of the variables studied did not follow a normal distribution, non-parametric tests were used. Results are presented as sample means and standard deviation. Non-parametric continuous quantitative variables were analyzed using the Kruskal-Wallis test or the U-Mann-Whitney test. Correlation analysis was performed using Rho Spearman test. Statistical significance was defined as p< 0.05. In addition, a stratified analysis by clinical subgroups was performed.

## Results

Characteristics of the study population

Among the 617 women recruited in the IPAQ study, losses to follow-up were detected for various reasons (spontaneous pregnancies, cancelled cycles, change of medical indication from IVF to intrauterine insemination), so finally 524 women were included for analysis. Regarding the accelerometer study, 105 women were provided with the device, of which 88 were eligible, as they wore the accelerometer for at least four days. There were three losses to follow-up, so the final number of included women for analysis was 85.

In the IPAQ population, the mean age of women was 34.8 years. Mean BMI was 24.67 kg/m² and mean AMH value was 2.57 ng/mL. In the accelerometer population, the mean age of women was 34.9 years and the mean BMI was 24.56 kg/m². Mean AMH value was 2.80 ng/mL (Table 1).

**Table 1:** Characteristics of ‘IPAQ population’ and ‘Accelerometer population’ undergoing IVF ᵃ 1 = low; 2 = moderate; 3 = high ᵇ 1 = light; 2 = moderate; 3 = vigorous; 4 = very vigorous Values are mean, median and SD unless stated otherwise. AMH: anti-mullerian hormone; BMI: body mass index; IPAQ: International Physical Activity Questionnaire; IVF: in vitro fertilization; MET: metabolic equivalent; MII: metaphase II oocyte; PA: physical activity; SD: standard deviation

Characteristics of the population	IPAQ (n=524)			Accelerometer (n=85)		
	Mean	Median	SD	Mean	Median	SD
Age (years)	34.8	36	3.63	34.9	35	3.17
AMH (ng/mL)	2.57	2.0	2.25	2.8	2.3	2.36
BMI (kg/m²)	24.67	23.47	4.6	24.56	23.28	4.64
Smoking (%)	26.5% (139/524)			23.5% (20/85)		
Number of retrieved oocytes	8.65	7.0	6.23	8.59	7.0	6.83
Number of mature oocytes (MII)	6.93	6.0	5.29	7.31	6.0	5.8
MET-min/week	2615.82	1836	2542.52	2563.2	2004.1	1612.9
PA level (MET-min/week) (1-3)ᵃ	2.15	2.0	0.69	-	-	-
PA level (counts-min/week) (1-4)ᵇ	-	-	-	1.63	2.0	0.628
Number of steps/day	-	-	-	9939.1	9355	3464
Sedentary time/day (hrs)	5.1	4.0	2.88	4.35	4.37	1.7

In regard to the etiology of infertility among the IPAQ population, the most frequent cause was male factor (26.3%), followed by homologous artificial insemination failure (18.9%). Endometriosis accounted for 12.4% of the sample, and ovulatory disorders for 5.3%. In the accelerometer substudy, distribution was similar. The most frequent cause was male factor (28.4%), followed by homologous artificial insemination failure (19.3%). Endometriosis accounted for 15.9% of the sample, and ovulatory disorders for 8%.

Regarding patients' distribution according to PA level, measured in MET, in the IPAQ population, 17.4% (n=91) of women were at the low PA level, 50.5% (n=265) at the moderate level and 32.1% (n=168) at the high level. The cut-off points were 600 and 3000 MET, which corresponded to percentiles p18 and p68, respectively. Groups did not differ in age, BMI, AMH, smoking or infertility etiology (Table 2).

**Table 2:** Characteristics of ‘IPAQ population’ split by clinical subgroups according to PA level Values are mean ± SD. *Statistical significance was defined as p < 0.05. Statistical analysis: Kruskal-Wallis or Fisher test. AMH: anti-mullerian hormone; BMI: body mass index; IPAQ: International Physical Activity Questionnaire; MET: metabolic equivalent; PA: physical activity.

IPAQ population	Low PA (<600 MET)	Moderate PA (600-3000 MET)	High PA (>3000 MET)	p-value
	(n=91)	(n=265)	(n=168)	
Age (years)	34.86 ± 3.52	34.9 ± 3.67	34.68 ± 3.66	p = 0.73
BMI (kg/m²)	24.52 ± 4.71	24.68 ± 4.63	24.71 ± 4.51	p = 0.911
AMH (ng/mL)	2.36 ± 2.34	2.67 ± 2.30	2.53 ± 2.13	p = 0.482
Smoking (%)	18% (25/139)	47.5% (66/139)	34.5% (48/139)	p = 0.679
Endometriosis	12.9% (8/62)	62.9% (39/62)	24.2% (15/62)	p = 0.161
Ovulatory disorders	18.8% (9/48)	54.2% (26/48)	27.1% (13/48)	p = 0.722

In the accelerometer population, taking the same cut-off points as a reference, 65.9% (n=56) of women were at the moderate PA level and 34.1% (n=29) at the high level. None of the patients (n=0) met the criteria for low PA level. The groups did not differ in age, AMH, smoking or infertility etiology but differed in terms of BMI, being of normo-weight in the moderate PA group and overweight in the high PA group (p=0.001) (Table 3). Of note, the correlation between IPAQ and accelerometer in MET was positive and statistically significant (p=0.031), although weak, with a correlation coefficient Rho Spearman r=0.23.

**Table 3:** Characteristics of ‘Accelerometer population’ split by clinical subgroups according to PA level Values are mean ± SD. *Statistical significance was defined as p < 0.05. Statistical analysis: Mann-Whitney U or Fisher test. AMH: anti-mullerian hormone; BMI: body mass index; MET: metabolic equivalent; PA: physical activity

Accelerometer population	Moderate PA (600-3000 MET)	High PA (>3000 MET)	p-value
	(n=56)	(n=29)	
Age (years)	34.46 ± 3.34	35.85 ± 2.58	p = 0.104
BMI (kg/m²)	22.41 ± 3.16	28.74 ± 4.2	p < 0.001*
AMH (ng/mL)	2.65 ± 2.34	3.1 ± 2.44	p = 0.278
Smoking (%)	65% (13/20)	35% (7/20)	p = 1
Endometriosis	78.6% (11/14)	21.4% (3/14)	p = 0.371
Ovulatory disorders	100% (7/7)	0% (0/7)	p = 0.09

Comparative analysis: association between PA and IVF outcomes

Globally, the number of retrieved oocytes was similar in all three groups of PA according to IPAQ (9.23 ± 7.72; 8.35 ± 5.57; 8.82 ± 6.38). Something similar happened with the number of mature oocytes (6.97 ± 5.99; 6.84 ± 4.85; 7.05 ± 5.61) (Table 4).

**Table 4:** Association between PA level in global ‘IPAQ population’ (n=524) and controlled ovarian stimulation outcome Values are mean ± SD. *Statistical significance was defined as p < 0.05. Statistical analysis: Kruskal-Wallis test. IPAQ: International Physical Activity Questionnaire; PA: physical activity.

	Low PA (n=91)	Moderate PA (n=265)	High PA (n=168)	p-value
Number of retrieved oocytes	9.23 ± 7.72	8.35 ± 5.57	8.82 ± 6.38	p = 0.85
Number of mature oocytes	6.97 ± 5.99	6.84 ± 4.85	7.05 ± 5.61	p = 0.81

When data were split regarding a number of clinical parameters (age, smoking, BMI, AMH), PA did not affect differently neither to retrieved nor mature oocytes. However, when stratifying by the etiology of infertility, there were statistically significant differences in the number of mature oocytes. In the subgroup of patients having endometriosis, the number of mature oocytes was significantly superior at high (8.40 ± 4.79) and moderate (7.71 ± 4.56) vs low PA level (3.63 ± 4.0) (p=0.024). In the subgroup of ovulatory disorders, there were also more mature oocytes at high (11.89 ± 5.42) and moderate level (8.0 ± 4.44) vs low PA level (3.5 ± 3.54) (p=0.038) (Table 5).

**Table 5:** Association between PA level in ‘IPAQ population’ (n=524) and number of retrieved or mature oocytes among clinical subgroups Values are mean ± SD. *Statistical significance was defined as p < 0.05. Statistical analysis: Kruskal-Wallis test. AMH: anti-mullerian hormone; BMI: body mass index; IPAQ: International Physical Activity Questionnaire; PA: physical activity.

IPAQ population	Number of retrieved oocytes				Number of mature oocytes			
	Low PA	Moderate PA	High PA	p-value	Low PA	Moderate PA	High PA	p-value
Age (37-40 years) (n=215)	6.94 ± 5.31	7.48 ± 5.20	9.39 ± 7.18	p = 0.115	5.85 ± 4.70	6.16 ± 4.49	7.58 ± 6.30	p = 0.246
BMI: normal (18.5-24.9 kg/m²) (n=304)	9.31 ± 8.39	8.51 ± 5.77	8.78 ± 6.90	p = 0.990	6.92 ± 6.26	7.06 ± 5.14	7.05 ± 6.18	p = 0.630
BMI: overweight (≥ 25 kg/m²) (n=244)	8.75 ± 7.14	8.15 ± 5.46	8.57 ± 5.38	p = 0.830	6.47 ± 5.71	6.53 ± 4.54	6.94 ± 4.81	p = 0.679
BMI: obesity (≥ 30 kg/m²) (n=90)	6.80 ± 5.16	7.88 ± 5.11	9.30 ± 5.06	p = 0.25	5.40 ± 3.31	6.06 ± 4.32	7.59 ± 5.54	p = 0.399
AMH < 1 ng/mL (n=115)	4.67 ± 3.45	4.41 ± 2.98	4.73 ± 3.26	p = 0.837	4.0 ± 3.34	3.67 ± 2.69	3.97 ± 3.19	p = 0.979
Smoking (n=139)	6.47 ± 4.44	8.0 ± 4.88	8.12 ± 5.74	p = 0.461	4.63 ± 3.18	6.46 ± 4.15	6.70 ± 5.65	p = 0.322
Endometriosis (n=61)	5.63 ± 4.69	9.24 ± 5.43	10.33 ± 5.11	p = 0.144	3.63 ± 4.0*	7.71 ± 4.56	8.40 ± 4.79*	p = 0.024*
Ovulatory disorders (n=26)	4.0 ± 4.24	9.8 ± 5.61	15.11 ± 6.49	p = 0.057	3.50 ± 3.54*	8.0 ± 4.44	11.89 ± 5.42*	p = 0.038*

In the analysis according to the accelerometer, there was globally no association between PA level (expressed in MET, counts, number of steps per day and sedentary behaviour (hours/day)) and ovarian response. When data were split regarding a number of clinical parameters (age, smoking, BMI, AMH), PA did not affect differently to oocyte number, neither retrieved nor mature (Table 6).

**Table 6:** Association between PA level in global ‘Accelerometer population’ (n=85) and controlled ovarian stimulation outcome Values are mean ± SD. *Statistical significance was defined as p < 0.05. Statistical analysis: Mann-Whitney U or Kruskal-Wallis test. MET: metabolic equivalent; PA: physical activity

	MET-min/week			Counts-min/week				
	Moderate PA (600-3000)	High PA (> 3000)	p-value	Light PA (<2690)	Moderate PA (2690– 6166)	Vigorous PA (6167– 9642)	Very vigorous PA (> 9643)	p-value
	(n=56)	(n=29)		(n=37)	(n=43)	(n=4)	(n=1)	
Retrieved oocytes	7.93 ± 5.38	9.93 ± 9.10	p = 0.506	8.49 ± 5.77	8.84 ± 7.72	6.75 ± 8.18	9.0	p = 0.721
Mature oocytes	6.79 ± 4.63	8.36 ± 7.64	p = 0.687	7.22 ± 4.99	7.42 ± 6.41	6.75 ± 8.18	8.0	p = 0.774
	Steps/day				Sedentary behaviour (hrs/day)			
	Low PA (<6949)	Moderate PA (6949– 10903)	High PA (>10903)	p-value	Low PA (>6.28)	Moderate PA (3.63– 6.28)	High PA (<3.63)	p-value
	(n=23)	(n=35)	(n=27)		(n=18)	(n=46)	(n=21)	
Retrieved oocytes	9.0 ± 5.92	8.19 ± 5.79	9.0 ± 8.89	p = 0.828	8.14 ± 4.93	8.69 ± 7.24	8.79 ± 7.31	p = 0.991
Mature oocytes	8.19 ± 5.50	6.70 ± 4.92	7.77 ± 7.27	p = 0.551	6.79 ± 4.41	7.45 ± 6.23	7.43 ± 6.0	p = 0.972

However, when stratifying by BMI, statistical significance was found. In normo-weight women, high PA level (expressed in MET) was significantly associated with a greater number of collected oocytes (20.83 ± 13.63) and mature oocytes (17.5 ± 10.78) compared to moderate PA level (7.56 ± 5.65; 6.4 ± 4.87 respectively) (p=0.005; p=0.004 respectively). When stratifying by infertility etiology, no significant differences were found in the subgroup of patients having endometriosis. More retrieved oocytes (9.33 ± 5.69) and more mature oocytes (7.33 ± 5.86) were obtained at high PA level compared to moderate level (6.73 ± 3.88; 6.0 ± 3.82 respectively), but without statistical significance (p=0.435; p=0.814 respectively). In patients with ovulatory disorders, it was not possible to perform the analysis due to a small sample size (n=7) and moreover because all women were at the same PA level (Table 7).

**Table 7:** Association between PA level (MET-min/week) in ‘Accelerometer population’ (n=85) and number of retrieved and mature oocytes among clinical subgroups Values are mean ± SD. *Statistical significance was defined as p < 0.05. Statistical analysis: Mann-Whitney U test. AMH: anti-mullerian hormone; BMI: body mass index; MET: metabolic equivalent; PA: physical activity

Accelerometer population		Number of retrieved oocytes			Number of mature oocytes		
		Moderate PA	High PA	p-value	Moderate PA	High PA	p-value
		(600 – 3000 MET)	(> 3000 MET)		(600 – 3000 MET)	(> 3000 MET)	
Age (37-40 years) (n=33)		6.71 ± 4.57	11.50 ± 10.41	p = 0.129	5.71 ± 3.89	9.69 ± 8.72	p = 0.181
BMI: normal (18.5-24.9 kg/m²) (n=49)		7.56 ± 5.65	20.83 ± 13.63	p = 0.005*	6.40 ± 4.87	17.50 ± 10.78	p = 0.004*
BMI: overweight (≥ 25 kg/m²) (n=36)		8.50 ± 4.52	6.95 ± 4.33	p = 0.219	7.33 ± 3.60	5.86 ± 4.12	p = 0.107
BMI: obesity (≥ 30 kg/m²) (n=13)		18.0 (n=1)	7.09 ± 5.67	p = 0.188	12.0 (n=1)	6.18 ± 5.46	p = 0.308
AMH < 1 ng/mL (n=14)		3.20 ± 2.04	7.25 ± 3.86	p = 0.085	2.80 ± 2.15	6.50 ± 3.11	p = 0.062
Smoking (n=20)		7.0 ± 4.86	5.67 ± 2.81	p = 0.741	6.0 ± 4.45	4.0 ± 2.37	p = 0.397
Endometriosis (n=14)		6.73 ± 3.88	9.33 ± 5.69	p = 0.435	6.0 ± 3.82	7.33 ± 5.86	p = 0.814
Ovulatory disorders (n=7)		7.29 ± 5.41	-	-	6.43 ± 4.79	-	-

## Discussion

There is a broad consensus on the positive impact of regular, aerobic, moderate-intensity PA on health. Benefits include improved insulin sensitivity, body weight control and the development of cardiovascular, respiratory, metabolic and immunological adaptations [1, 27]. However, it is unclear whether PA could produce any local benefits in the ovary that could increase the number of total or mature oocytes and therefore improve the outcome of an IVF cycle.

In the present study, global PA assessed by IPAQ or by accelerometer did not influence ovarian response to stimulation in the IVF cycle. However, in certain clinical subgroups (endometriosis, ovulatory disorders or normal BMI), high PA was significantly associated with a superior ovarian response and/or better oocyte quality. High PA occurred mainly in those patients who practiced regular, planned and structured PA, i.e., ‘physical exercise’, which generates anatomical and functional adaptations after several weeks if a certain threshold of PA is reached. This threshold has not yet been established in relation to IVF.

Our study has been primarily aimed at assessing the influence of PA on ovarian response, whereas some of the previous research focused mainly on pregnancy rate [9-13]. This may explain some of the discrepancies, as a higher pregnancy rate may be due to a better oocyte factor or superior endometrial receptivity. In this study, we chose to focus on the oocyte factor as it is easily measurable, objectively. Analyzing pregnancy rates involves, on the one hand, consideration of the endometrial factor, whose independent assessment is conceptually and methodologically complicated. Unquestionably, the best way to measure both ovarian and endometrial response is through pregnancy rates. However, this brings with it the difficulty of including the male factor, which should not be associated with women's PA.

In previous studies that referred to pregnancy rate in IVF in relation to PA, some authors found no association or reported a negative effect of PA [6,8,28,29], although most authors found a statistically significantly higher pregnancy rate in regular PA groups [2,5,7,10,13]. Of the three authors who also analyzed the oocyte factor, Söritsa and Prémusz found a higher number of retrieved oocytes in relation to PA, in contrast to Ferreira, who found no difference [3,10,11]. Only Prémusz analyzed oocyte quality, reporting more mature oocytes in the moderate PA group. Speculation could be made as to whether PA has a positive impact on the endometrium or on the oocyte. Regarding the endometrium, it could increase vascular flow or act as an immunomodulator, favoring embryo implantation; although two studies in which PA was measured by accelerometer from embryo transfer to pregnancy test found no significant differences in pregnancy rate between active and sedentary women [2,11]. Two meta-analyses have explored the impact of PA on IVF cycle outcomes [12,13]. Rao concluded that PA for more than 2.5 hours per week prior to IVF/ICSI cycle significantly improved clinical pregnancy and livebirth rates [12]. Subsequently, Kakargia reported that PA prior to the IVF/ICSI cycle improved clinical pregnancy rate, although the authors found no significant differences in implantation, miscarriage or livebirth rates [13].

In the present study, a superior ovarian response and/or higher number of mature oocytes was observed in certain clinical subgroups (endometriosis, ovulatory disorders and normal BMI). This suggests that certain patient profiles may be candidates for PA as part of a customized therapeutic strategy. It seems reasonable to suppose that, just as different clinical parameters have a different impact on IVF outcomes, perhaps PA could have a differential impact according to coexisting clinical variables (maternal age, ovarian reserve, smoking, obesity, infertility etiology, etc.). Of these clinical parameters, the one that has received the most attention is obesity/overweight, due to its clear inverse association with PA.

Scientific evidence shows a worse response to stimulation, poorer oocyte quality and lower clinical pregnancy rate in obese women. It has been suggested that obesity induces chronic low-grade systemic inflammation. In the present study, there was no association between ovarian response and PA level, as reported by Moran or Kiel [28,30]. Palomba, on the contrary, reported better IVF cycle outcomes in obese women who performed regular PA [7].

In women with polycystic ovarian syndrome, moderate PA is recommended in the treatment of this pathology and has been associated with increased spontaneous pregnancy and live birth rates [14]. In the present study, by IPAQ, significantly more mature oocytes were collected at the high PA level.

As for endometriosis, it has been reported that a suboptimal microenvironment in the follicle, where a proinflammatory phenotype predominates as well as oxidative stress, would negatively affect oocyte cytoplasmic competence [17]. In the present IPAQ study, significantly higher numbers of mature oocytes were obtained at the high and moderate PA level vs low PA level.

Among the strengths of this study, it should be noted that it was the second in terms of sample size after Morris [6], using questionnaires as a method for assessing PA. Concerning the validated specific IPAQ questionnaire, this study was the one with the largest sample size. Furthermore, it was the one with the largest sample size performing a double analysis, IPAQ and triaxial accelerometer. The objective assessment of PA can be considered a strength of the study, as well as its prospective design.

Secondly, an analysis by clinical subgroups was performed allowing to analyze the influence of PA in different clinical conditions. It must be highlighted that, as a consequence of the many clinical subgroups considered, our study could have obtained, by chance in some cases, p-values < 0.05. It is well known that, when performing multiple hypothesis tests, the probability of making one or more false discoveries, or type I errors, increases.

There are some limitations to this study, so results should be interpreted with caution. Firstly, although using a validated questionnaire - as IPAQ - to assess PA, this method has some disadvantages, such as subjectivity, recall bias or overestimation of performed PA. On the other hand, accelerometers show the inconvenience of having to be removed during aquatic activities or presenting a surveillance bias. Secondly, this was an observational study. Despite taking into account some confounding factors, it is possible that high PA may be associated with certain parameters that, in themselves, could be related to a better outcome of IVF cycle, such as healthy lifestyle habits, diet, higher socioeconomic status and lower adiposity, among others. Thirdly, the sample size after stratification was small, especially in the accelerometer study.

## Conclusions

PA has multisystemic benefits for human health. Evidence so far is insufficient to draw robust conclusions about the impact of PA on IVF outcomes.

In the present study, differences in short-term PA among women undergoing IVF cycles had globally no influence on ovarian response, defined as the number of retrieved and mature oocytes. However, in certain subgroups of patients (endometriosis, ovulatory disorders, normal-BMI), high PA was associated with a superior ovarian response, showing an increased number of mature oocytes. These findings open up new lines of research in relation to PA, taking into account patients’ underlying conditions and etiology of infertility. More prospective clinical studies are needed, specifically in these subgroups.
